# Experiences of Community Members and Health Workers Regarding Malaria Control Programmes in the Rural Ingwavuma Community, uMkhanyakude District Municipality, KwaZulu‐Natal Province, South Africa

**DOI:** 10.1002/puh2.70132

**Published:** 2025-11-08

**Authors:** May Thulisa Thembakazi, Chikafu Herbert, Khuzwayo Nelisiwe

**Affiliations:** ^1^ Discipline of Public Health, School of Nursing and Public Health University of KwaZulu‐Natal Durban South Africa

**Keywords:** indoor residual spraying, KwaZulu‐Natal, malaria control and elimination, malaria endemic, South Africa

## Abstract

**Background:**

Malaria remains a health challenge in South Africa, particularly in Limpopo, Mpumalanga and KwaZulu‐Natal, where elimination measures are ongoing. This study explored the experiences of community members and healthcare workers with malaria control programmes in Ingwavuma, a rural community in the uMkhanyakude district bordering Mozambique.

**Methods:**

We used an exploratory qualitative design. Data were collected through eight in‐depth interviews (IDIs) with healthcare workers (a supervisor, a senior administrator and fieldworkers) involved in malaria control and eight focus group discussions (FGDs) with 88 community participants. IDIs were conducted at a local clinic, whereas the FGDs were held in six villages using pre‐tested discussion guides in isiZulu. All sessions were audio recorded, transcribed and translated from isiZulu to English. Data were analysed thematically.

**Findings:**

Community members generally viewed malaria control activities positively, particularly health education, indoor residual spraying (IRS) and vector surveillance. Rapport between malaria control personnel and communities was strong, with respectful engagement that enhanced programme acceptance. However, healthcare workers highlighted challenges in malaria surveillance linked to illegal cross‐border movement between South Africa and Mozambique. Local social protests occasionally disrupted spraying activities, with a few communities blocking access to their homes. Some participants reported negative experiences with IRS.

**Conclusion:**

Community‐based malaria control measures have been instrumental in preventing malaria. Our findings indicate a need for continuous community engagement with all stakeholders to maintain good rapport with communities. The adverse experiences attributed to IRS require further investigation.

## Graphical abstract

Community and healthcare worker perspectives reveal strong acceptance of malaria control programmes in the rural settings of South Africa, though challenges persist with cross‐border movement, local protests and negative spraying experiences. Strengthening community engagement remains critical for sustaining malaria elimination efforts.

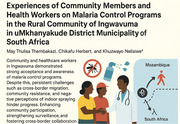

## 1 Background

Malaria remains a major public health problem in Africa despite being preventable and treatable [1, 2]. It disproportionately impacts socially vulnerable populations, including infants and pregnant women, resulting in poor health outcomes. In sub‐Saharan Africa, which suffers from a high incidence of communicable diseases, malaria is a major cause of premature deaths and strains health systems through increased hospitalisations [3, 4]. Malaria also has significant socioeconomic impacts, particularly in low‐income households from high treatment costs, decreased productivity in rural subsistence farming, and income loss due to illness‐related work absence [5]. The persistent burden of malaria in sub‐Saharan Africa is attributed to multiple factors, including climate change, declining funding and suboptimal implementation of essential interventions [6, 7].

In South Africa, malaria transmission is seasonal and remains endemic in some districts of Limpopo, Mpumalanga and KwaZulu‐Natal Provinces [8, 9]. These provinces share borders with neighbouring malaria‐endemic countries, influencing malaria transmission dynamics [3]. However, the burden of malaria varies widely across and within these endemic provinces. During the 2022–2023 malaria seasons, the Vhembe and Mopani districts in Limpopo Province accounted for 82% of reported malaria cases, whereas the Ehlanzeni district in Mpumalanga Province contributed 87% to the province's case burden. KwaZulu‐Natal recorded the lowest malaria incidence among the three provinces, though transmission was unevenly distributed. uMkhanyakude, King Cetshwayo and Zululand districts contributed 33%, 20% and 4% of the provincial cases, respectively, whereas the non‐endemic eThekwini district represented 25% of the province's burden [9, 10].

South Africa has implemented various strategies to control and eliminate malaria, leading to positive outcomes [10]. Key components of the strategies include active surveillance of confirmed cases and prompt treatment to interrupt local transmission [11]. The other strategies include using sensitive diagnostic tests to detect low‐level parasitaemia, which is crucial in this effort, as it maintains a high level of malaria awareness among both communities and health workers, even as the prevalence of the disease decreases [1]. However, the decrease in malaria cases related to chemical parasite control and improved access to treatment is a significant milestone; a noteworthy increase in the risk of parasite drug and vector resistance requires continuous monitoring [12, 13, 14]. Furthermore, eliminating malaria transmission requires collaborative regional strategies to address the spread of malaria and ensure that control measures are uniformly effective across international borders, as cross‐border transmission can undermine local malaria control efforts. Sustaining progress in malaria control requires continued resourcing, even in the face of reduced case numbers [15]. Ongoing investment in health infrastructure, surveillance systems and community education is vital to prevent resurgence.

Although systemic interventions are crucial for interrupting malaria transmission, the success of control programmes in endemic areas also depends on social and programmatic factors. Social factors, such as community awareness, cultural practices and local health beliefs, significantly influence how prevention and treatment measures are received and adopted. Programme‐related factors, including the accessibility and quality of healthcare services, the training of health workers and the availability of resources, directly impact the success of malaria control initiatives [10, 16, 17]. Effective malaria control and elimination programmes must consider these elements to ensure that interventions are practical and sustainable within the local context. Integrating a deep understanding of these social and programme‐related factors with systematic approaches can enhance the overall efficacy of malaria control efforts. This holistic strategy ensures that interventions are scientifically sound, culturally appropriate and operationally feasible, leading to more successful and enduring malaria eradication outcomes [17, 18]. This qualitative study explored the experiences of community members and healthcare workers regarding malaria control intervention in the uMkhanyakude District Municipality in KwaZulu‐Natal Province, where malaria remains endemic despite the implementation of malaria control programmes.

## 2 Methodology

### 2.1 Study Area

The study was conducted in the Ingwavuma community, a rural area in the uMkhanyakude District Municipality in the northeastern part of KwaZulu‐Natal Province in South Africa. The uMkhanyakude District Municipality is ecologically diverse, stretching from the coastal areas on the Indian Ocean to the country's international borders with Mozambique and Eswatini. uMkhanyakude has five local municipalities, including Jozini, with 20 municipal wards. The study area is along the borders of Eswatini and Mozambique and falls under the traditional authority of the Mathenjwa chieftaincy. The population in the Ingwavuma community is predominantly Black African, and the majority speaks isiZulu [19].

The Jozini Local Municipality is predominantly rural and socially vulnerable. The local economy is primarily subsistence‐based, with most households relying on small‐scale rainfed agriculture and social grants for livelihood, amid high unemployment rates [20, 21]. Erratic rainfall (600–1000 mm annually) impacts agricultural productivity and food security, with only 26% of households reporting having sufficient food stocks year‐round [21, 22, 23]. Educational attainment is low, with less than half of adults completing secondary education [21, 24]. Furthermore, access to basic services is constrained: many households lack piped water and adequate housing infrastructure. Although healthcare services are publicly funded, their utilisation is hindered by long travel distances and transport costs, particularly in remote areas [21, 25, 26]. Although micro‐level epidemiological data on malaria in Jozini are scant, the municipality is classified as a high transmission zone [27, 28] with *Anopheles arabiensis* the dominant malaria vector [29]. A morbidity survey conducted in the municipality reported a low prevalence (2%) in a sample of 2094 participants, with most cases imported from Mozambique and concentrated in a border region [30].

### 2.2 Study Design

This qualitative explorative study aimed to document the experiences and perceptions of malaria control in a socially vulnerable rural community in South Africa.

### 2.3 Study Population, Sampling and Sample Size

The study's target population included healthcare workers actively engaged in malaria control programmes and community members from the Ingwavuma area. Purposive sampling was used to select key informants with direct experience in malaria control activities. Community members were selected from households participating in a malaria‐related knowledge, attitudes and practices (KAP) survey we conducted before this qualitative study. We used a systematic approach to ensure representation across wards included in the KAP survey. Ninety‐two participants were recruited, including 88 community members (18 years and older) and four key healthcare worker informants who contributed to in‐depth interviews (IDIs).

### 2.4 Data Collection

A combination of qualitative data collection methods was used to comprehensively understand community and health worker perspectives and experiences regarding malaria control programmes. These included focus group discussions (FGDs) and IDIs. FGDs were conducted with community members to explore their views and experiences. FGDs were conducted with community members to gather their views and experiences. These discussions took place in venues strategically chosen to minimise participants’ travel time and ensure they did not disrupt their livelihood activities. Scheduling the FGDs at times that were convenient for the participants. An experienced facilitator is proficient in the local isiZulu dialect and moderated each FGD to ensure rich and meaningful discussions. In addition, FGDs were stratified by age group and gender to encourage open and robust discussions, as conservative cultural norms may have restricted the expression of viewpoints in the presence of older people or individuals of different genders. Each FGD lasted approximately 1 h to maintain participants’ concentration and participation.

Four IDIs were conducted with key informants. The interviews included the MCP supervisor based at the municipal administrative station, a senior administrator of a local clinic and community health workers working within the MCP at the two clinics in the study area. IDIs were conducted in isiZulu with community health workers and in English with the clinic manager and MCP personnel. This language choice was based on the preferences and language proficiency of the interviewees. The interviews were scheduled at the most convenient times for the participants and took place in comfortable and confidential settings to encourage open and honest responses. The FGDs and IDIs were recorded using a digital handheld voice recorder.

### 2.5 Ethical Considerations

The Human and Social Sciences Research Ethics Committee (HSSREC) of the University of KwaZulu‐Natal granted ethical clearance and permission to conduct the study (HSSREC/00002562/2021). Permission to access the area to conduct the proposed study was requested from all relevant gatekeepers, including traditional leaders of the study areas and the Department of Health. An outline of the study was presented to consenting potential adult participants (aged at least 18 years), explaining everything about the study, after which consenting participants signed consent forms. Participation in the study was voluntary, and participants were informed of their right to withdraw from the study at any time without prejudice. Permission to audio‐record and scribe during data collection was sought and granted before interviews and FGDs. We ensured confidentiality during the discussions through the use of codenames.

### 2.6 Data Analysis

The audio data were transcribed verbatim and translated into English by a qualified, experienced translator to facilitate analysis. Thematic analysis was used to analyse the data. The analysis followed a systematic process, starting with immersion into the data, where the researcher thoroughly read and reread the transcripts, listened to the audio recordings and took detailed notes to achieve a deep understanding of the data to identify broad themes and patterns in the data. After immersion, the researcher coded the data, identified keywords and phrases representing themes and then grouped them into themes and emergent subthemes.

### 2.7 Trustworthiness

The researchers ensured the trustworthiness of the results by adhering to the four criteria proposed by Lincoln and Cuba [31]. Credibility was achieved by engaging with community members and health workers over an extended period, using focus groups and IDIs and conducting member checks. Transferability was attained by providing detailed descriptions of the community setting, the health workers’ environment and the malaria control programmes. Dependability was ensured by maintaining a detailed record of the research process and having colleagues review the process and findings. Confirmability was achieved by the first author documenting reflections on potential biases and seeking participant feedback to ensure the findings reflect their experiences.

## 3 Results

### 3.1 Socio‐Demographic Characteristics of the Participants

Eighty‐eight participants contributed to the eight FGDs, with group sizes ranging from 7 to 11 participants. We also conducted IDIs with four healthcare workers. The study had 92 participants, comprising 53 (57.6%) females and 39 (42.4%) males, as shown in Tables 1 and 2.

**TABLE 1:** Socio‐demographic characteristics of focus group discussion (FGD) participants.

FGD	Age (year)	Gender	Role in the community	Number of respondents
FGD 1	28–34	Males (4), females (8)	Community members	12
FGD 2	21–32	Males (5), females (6)	Community members	11
FGD 3	25–43	Males (5), females (7)	Community members	12
FGD 4	20–35	Males (4), females (5)	Community members	9
FGD 5	19–41	Males (5), females (7)	Community members	12
FGD 6	22–47	Males (4), females (5)	Community members	9
FGD 7	18–36	Males (5), females (6)	Community members	11
FGD 8	18–39	Males (5), females (7)	Community members	12
Total				88

**TABLE 2:** Socio‐demographic characteristics of in‐depth interview participants.

Age (year)	Gender	Role in the community	Number of respondents
48–52	Male	Healthcare worker	1
27–30	Male	Malaria control programme supervisor	1
25–28	Female	Malaria fieldworker	1
50–55	Female	Community healthcare worker	1
Total			4

### 3.2 Main Results

Four themes emerged from the data analysis, indicating the nuanced nature of malaria control in the Ingwavuma community. Participants spoke positively about primary prevention strategies such as health education and indoor residual spraying (IRS), recognising their role in reducing transmission. They also acknowledged the importance of community surveys and surveillance, though these efforts are hampered by cross‐border movement and limited local involvement. Strong trust in MCP staff, built on respectful engagement and consistent communication, emerged as a key enabler of programme acceptance. However, significant implementation challenges persist, including financial constraints, adverse experiences with spraying and social grievances from the community.

## 4 Primary Prevention Strategies

Participants highlighted various primary prevention strategies, including health education and IRS. Health education efforts aimed to raise awareness about malaria transmission, prevention methods and seeking timely treatment. IRS was recognised as a crucial intervention for reducing mosquito populations and preventing malaria transmission within homes and communities.

### 4.1 Health Education

Health education emerged as a key strategy to eliminate malaria in the Ingwavuma community. Participants indicated that the MCP's Information, Education and Communication (IEC) team conducted these health education initiatives. The IEC team provided malaria education across the uMkhanyakude district, conducting health promotion sessions in schools, clinics, community halls and other accessible venues strategically located to ensure accessibility for community members. The MCP supervisor confirmed as follows:

The IEC team specifically conducted health education in all areas of uMkhanyakude, in schools, clinics, and communities, to ensure that the necessary information reached all people in the right places and at the right time. (IDI participant 1)

Participants reported receiving detailed information from healthcare workers when asked about health education activities. They learned about malaria symptoms and prevention methods. A community member in Ward 13 remarked:

The healthcare workers from the Malaria Centre and the clinics are sent to teach us about malaria, its symptoms, how it is transmitted, and the areas where it is more likely to occur. They also teach us about cleanliness to prevent malaria in the community. (FGD participant 2)

A community health worker corroborated this account:

We mobilise them (communities) by going door‐to‐door, informing them of the date and when to meet for health education. We usually meet on the playgrounds or in the community halls. We facilitate these meetings through community meetings, schools, clinics, and police stations. (IDI participant 3)

### 4.2 Indoor Residual Spraying

We found that the IRS team actively worked in the community. Although most IRS activities occur during the summer when malaria transmission is highest, the IRS team remains vigilant year‐round. They also respond to malaria cases that arise out of season. This ensures continuous protection and swift action against malaria transmission. A participant from the IRS team had this to say.

The spraying season is always the last quarter of the year. If there is a local case in the middle of the year, around June, the team performs a focus spraying, meaning they spray only the area with malaria cases. (IDI participant 2)

In addition to the above, another participant from the IRS team also explained that the team's work followed emergent needs and worked to prevent further transmission.

The team conducts mass screening, which means members screen and test everybody who is 2 km away from the index house; the index house is where there is a malaria case. (IDI participant 3)

#### 4.2.1 Field Observations

**FIGURE 1:**
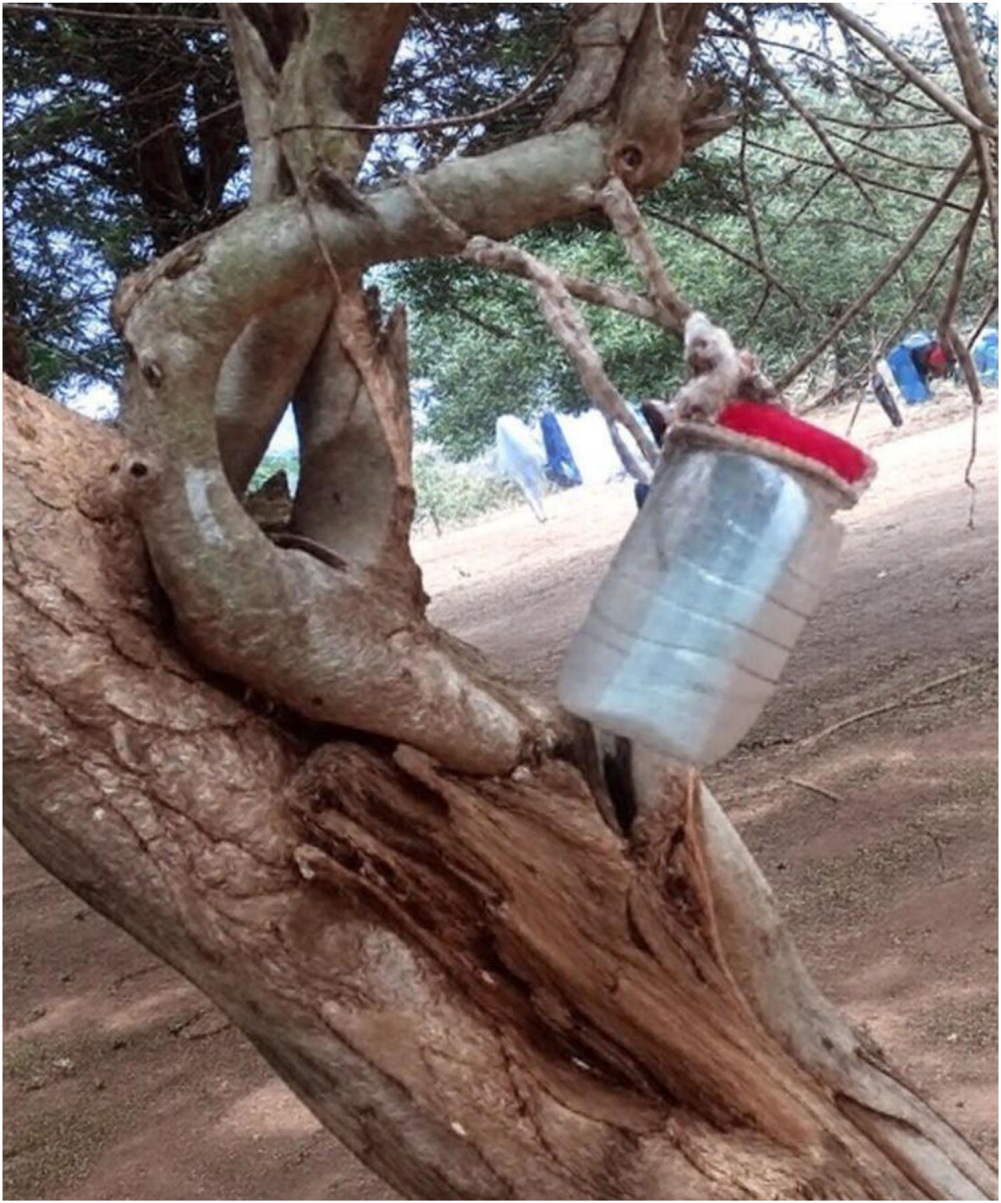
A transparent container with a household's IRS record hung on a tree within the homestead.

We did not witness any IRS during our data collection. However, we could glean some of the IRS team's operations from IRS records kept at households. A paper‐based schedule is kept at all households receiving IRS and at an accessible spot within the homestead, often secured in a transparent plastic container hung on a tree (Figure 1) or on the roof eaves or latched to a wall. MCP fieldworkers recorded when respective homes received the IRS and signed the card. We noted recently sprayed homesteads from household‐based records, as some of the data were collected during the IRS season, except for some areas in Ward 13, where the MCP was denied home access due to political constraints. Most IRS cards had signed entries from the previous year in these areas, even in areas we surveyed post the IRS season.

## 5 Views on Community Surveys

Community members and health workers shared their perspectives on the role and effectiveness of community surveys in malaria control programmes. These surveys were seen as valuable tools for gathering data on malaria prevalence, identifying high‐risk areas and tailoring interventions to meet the specific needs of different communities. Participants discussed the importance of regular and comprehensive surveys for programme planning and evaluation.

### 5.1 Active Surveillance

The study results indicate active surveillance has significantly contributed to malaria control and elimination since 2017. The MCP supervisor highlighted the critical role of monitoring migration routes to contain imported malaria cases, particularly from illegally crossing the Mozambique border into South Africa. Notwithstanding its contribution to reducing cases, the MCP supervisor also detailed challenges to active border surveillance in the border region.

Since 2017, surveillance has contributed to malaria control in this area. However, illegal border crossings have presented a challenge since they are undocumented surveillance areas. We use rapid diagnostic tests (RDTs) to test malaria symptoms in a person, taking blood spots and slides for laboratory analysis, ensuring that everyone is screened, tested, and treated to minimise the chances of transmission, and conducting active surveillance at least daily. Still, now, with illegal borders, it is not easy to test and treat undocumented people. (IDI participant)

When asked how they execute malaria surveillance in illegal border crossing points, a field worker from the MCP responded as follows:

We are grouped into two groups, A and B. Group A is at the illegal borders around the Manguzi area, and Group B is at the illegal border crossings around the Jozini area to see and control the situation of cross‐border movements regarding malaria. We are looking forward to the elimination phase of zero local cases. We are trying very hard to minimise the flocking of people from the countries they move up and down. (IDI participant)

The clinic administrator responded thus:

Active surveillance has been identified as a great success so far because several local cases have decreased since 2017. It is a good sign that active surveillance limits the chances of malaria transmission. Another thing that has been identified is the number of imported cases from illegal borders, most of which are from Mozambique, meaning they have successfully identified cases. Moving into illegal borders to South Africa, they educate them first about malaria‐related information, test, and treat them immediately if they are malaria‐positive because they have a professional nurse. Suppose they cannot treat the case due to their conditions. In that case, they write a referral letter to the clinic or hospital to treat them according to the doctor's supervision, such as when an individual is mentally disturbed due to severe malaria. (IDI participant 4)

### 5.2 Entomological Survey

The MCP supervisor noted that breeding sites were checked and identified through the entomological survey to identify the type of mosquitoes available in the area. In addition, the outcome of the entomological survey was identified as a critical factor in implementing the IRS, notably for hotspot detection and strategic planning of the IRS.

The Malaria Control Programme supervisor explained the duties of the team, thus:

We also have an entomological team that conducts an entomological survey; when they do an entomological survey, they go to the breeding sites, check the larvae, and then write a report on what they have found. They must respond with a larva survey if they have found the larva, the targeted interventions, or the measures. The entomology team goes to those areas and conducts the entomological surveys. They bring the report to the environmental health practitioners so that when they plan for the following IRS season, they know which areas to focus on because they have larvae that are considered high‐risk or hotspots. Some areas are dry and do not need IRS because there is no malaria, which also helps guide the IRS team. The number of malaria cases imported into that area is essential. There is a local transmission; they look at the type of mosquito that transmits the parasite Plasmodium falciparum. (IDI participant)

We also explored the involvement of community members and the MCP field workers in the entomological survey. Our findings revealed that neither community members nor the field workers were actively engaged in the process. The field worker explained that she lacked the necessary knowledge to conduct an entomological survey as it was outside her expertise. She also provided insight into why community members were not involved, explaining:

I do not do the entomological survey. It is done by people trained for that job, and another reason why it is done at night is that community members are not involved. (IDI participant)

Although community members needed to learn the explicit term for an entomological survey or the processes involved, they revealed that they would call on the MCP field worker to come for observations whenever they saw many mosquitoes in their area. A female participant from Ward 17 commented:

When we have a problem with mosquitoes, we call the people from malaria to come and spray; they come and check our place first. If they see a need to spray, they do so. (FDG participant)

## 6 Attitudes of Community Members Toward the Malaria Control Programme Staff

According to community members, the MCP staff and healthcare workers demonstrated respect and consideration in their activities, fostering a positive relationship with the community. One participant from Ward 13 remarked that the respectful approach and thoroughness of the MCP staff had significantly enhanced community trust and engagement, leading to greater participation in malaria prevention efforts and a more effective overall programme.

They treat us well; they spray our houses before the rainy season because malaria is expected in the rainy season. They tell us in advance before they come and spray our homes, and give us more information about malaria. They screen and test us to see if we have malaria or not. We have a good relationship with them; they keep their appointments when they promise to visit our homes. (FGD participant)

Similarly, a community member from Ward 15 thought that healthcare workers did well performing their jobs and relating to the community. This is how he responded:

What can I say? Do we have a good relationship with the healthcare workers? They always tell us what to do and what to use when it is rainy, such as closing the windows before sunset, before six o'clock. They spray nicely, and we no longer have malaria in our area, except for a few people coming from the illegal borders of Mozambique. (FGD participant)

## 7 Challenges Related to Intervention Implementation

The results indicate that several challenges persist while malaria elimination interventions implemented in the Ingwavuma community between 2014 and 2020 were broadly accepted. These include financial constraints and community resistance to indoor spraying. Additionally, community health workers highlighted that a key obstacle in malaria prevention efforts was their limited ability to influence the movement of community members, particularly across borders, which hindered the effectiveness of control measures.

### 7.1 Financial Constraints

Household financial difficulty attributed to unemployment impacts malaria control among the vulnerable in Ingwavuma. Some community members who live far from healthcare facilities indicated that financial constraints were linked to the delay in seeking care whenever they suspected malaria symptoms. Sometimes, they walk long distances to seek care. Furthermore, financial constraints render vulnerable households unable to purchase mosquito repellents and resort to burning cow dung and toilet paper to repel mosquitoes instead of conventional repellents such as mosquito coils. The participants noted the following:

We cannot afford mosquito repellents because we are not working. (FGD participant)

I do not use anything because I do not work, and I have to buy food with the little money I get. (FGD Participant)

The challenge is that we cannot afford things like mosquito coils and nets because we are not working. (FGD participant)

Another problem is that I stay far from the clinic; even if I suspect something, I cannot go immediately because sometimes I do not have money for a taxi. (FGD participant)

### 7.2 Adverse Experiences With Mosquito Repellents

Participants reported various adverse experiences attributed to mosquito control interventions, particularly IRS administered during the malaria control programme. It was reported that these IRS agents often cause discomfort, with some participants reporting allergic reactions, skin irritations and respiratory problems. The negative experiences with IRS led to a decrease, causing some households to dislike a critical malaria control intervention and promoting alternative interventions.

The things (mosquito repellents) they use have a bad smell; some of us have sinuses. (FGD participant)

The programme workers were also accused of being untidy.

After spraying our houses, they leave our clothes dirty with something white. (FGD participant)

However, the other area of Ward 13 had a different view of the services provided by MCP. Their response was linked to the political constraints of the employment processes.

We stopped them from spraying our homes this season because they employ people from other wards and municipalities. So, we said we did not want to see them again in our homes. (FGD participant)

## 8 Discussion

Although South Africa has implemented various strategies to eliminate malaria, it remains a public health concern in three provinces [8, 11, 30, 32, 33]. The burden is concentrated in poor rural communities, with most cases linked to cross‐border transmission from neighbouring countries [1, 34, 35]. This study explored community members’ and healthcare workers’ attitudes and experiences with malaria control efforts in the uMkhanyakude District Municipality. Overall, we found largely positive perceptions of interventions, particularly regarding health education, surveillance and IRS. These findings align with previous studies in South African provinces [1, 36] and sub‐Saharan Africa [37, 38, 39].

Our findings reflect pillars of the Malaria Elimination Strategic Plan and the National Vector Control Strategy that emphasise health promotion, vector control, surveillance and locally responsive, data‐driven approaches as foundational to malaria elimination [32, 33]. Health education emerged as a key point of alignment, with participants acknowledging the role of IEC teams in promoting preventive behaviours. Resultantly, study participants demonstrated a strong awareness of primary prevention programmes aimed at controlling and eliminating malaria transmission within their communities. Similarly, active surveillance was recognised, including along informal border crossings that have been major drivers of transmissions in uMkhanyakude [30]. These ideal attributes of effective programming for reaching the target population are noteworthy, particularly health education, given its crucial role reported in malaria control programmes in southern Africa [34, 35].

The community was not directly involved in entomological surveys. However, community members reported areas with dense mosquito swarms to MCP officials, indicating an awareness of malaria risk factors attributable to practical health promotion efforts. These community‐initiated reports also reflect a positive attitude toward the programme and a strong rapport between the community and MCP. Despite this, worrisome implementation challenges regarding the IRS persist in the Ingwavuma community. Although IRS remains the primary household‐based vector control method in South Africa, some areas resented it due to its perceived side effects and in protest to socio‐political grievances over social service delivery and limited economic inequity, expressed through boycotting IRS. Service protests are a common expression of community displeasure, often expressed in various forms in South Africa, including rural uMkhanyakude [36, 37, 38]. However, the unfortunate inadvertent consequence of the socio‐political contestations may be the sustainment of vector and infection reservoirs that perpetuate transmission and undermine malaria elimination efforts [39]. The disruptions in malaria elimination efforts are exacerbated by limited use of mosquito repellents and delays in seeking care for suspected malaria illness. Community participants ascribed delayed health seeking to distant facilities and financial constraints consistent with evidence from earlier studies [21, 25, 26].

Insecticide‐treated bed nets are not part of the malaria elimination strategy in South Africa [32]. Therefore, unsurprisingly, ITNs are not used in the rural community of Ingwavuma. However, KwaZulu‐Natal has experienced extreme heat and precipitation cycles in the new millenniums that, in addition to the region's humid climate, provide conducive conditions for the breeding of malaria vectors [1, 39, 40, 41]. In the backdrop of resurgent infections causing the postponement of targeted elimination timelines in the remaining endemic areas nearing elimination [42], insecticide‐treated nets may be a worthwhile consideration in the malaria elimination strategy in hotspots irrespective of infection levels. All other things remaining the same, insecticide‐treated nets would supplement protection from IRS and sustain gains from IRS and other measures [42].

### 8.1 Limitations and Strengths

We report a study design‐related limitation. This study's cross‐sectional exploratory qualitative design limited its ability to capture changes in perceptions and experiences over time. However, it offers valuable strengths by providing micro‐level insights into community and health worker perspectives, often overlooked in studies reliant on health records and household surveys. The insights from qualitative enquiries are crucial for refining and adapting malaria control and elimination strategies through participatory modelling.

## 9 Conclusion

This study explored malaria control experiences among community members and health workers in the rural Ingwavuma community in northeastern KwaZulu‐Natal, South Africa. From the participants’ viewpoint, malaria control interventions have reduced malaria cases. The community was satisfied with some of the interventions. However, concerns regarding irregular access to some sections of the community and misconceptions about the safety of IRS remain, affecting the efficacy of malaria elimination measures. We have two recommendations. First, community engagement and collaboration between policymakers, programme implementers and community stakeholders, including community leaders, are needed to strengthen rapport. Second, there is a need for continuous health education to enhance awareness of malaria. Improving rapport and malaria awareness in the Ingwavuma community may enable universal IRS and enhance malaria elimination.

## Author Contributions

May Thulisa Thembakazi conceptualised the project and collected and analysed the data. In addition, she wrote the first draft of the manuscript. Chikafu Herbert and Khuzwayo Nelisiwe reviewed and approved all the drafts of the manuscript.

## Conflicts of Interest

The authors declare no conflicts of interest.

## Data Availability

Data derived from this study are available on written reasonable request from the lead author.
